# Evaluation of AC(n) and C(-106)T polymorphisms of the aldose reductase gene in Brazilian patients with *DM1* and susceptibility to diabetic retinopathy

**Published:** 2007-05-23

**Authors:** Flávio Richeti, Renata Maria Noronha, Ricardo Temudo Lessa Waetge, José Paulo Cabral de Vasconcellos, Osías Francisco de Souza, Bianca Kneipp, Nilma Assis, Mylene Neves Rocha, Luís Eduardo Procópio Calliari, Carlos Alberto Longui, Osmar Monte, Monica Barbosa de Melo

**Affiliations:** 1 Laboratory of Molecular Medicine, Department of Physiology, Faculty of Medical Sciences, Irmandade da Santa Casa de Misericórdia de São Paulo, Brazil; 2 Pediatric Endocrinology Unit, Department of Pediatrics, Irmandade da Santa Casa de Misericórdia de São Paulo, Brazil; 3 Retina Service, Department of Ophthalmology, Irmandade da Santa Casa de Misericórdia de São Paulo, Brazil; 4 Department of Ophthalmology, Faculty of Medical Sciences, State University of Campinas São Paulo, Brazil; 5 Department of Medical Genetics, Faculty of Medical Sciences, State University of Campinas São Paulo, Brazil; 6 Endocrinology Discipline, Department of Clinical Medicine, Irmandade da Santa Casa de Misericórdia de São Paulo, Brazil

## Abstract

**Purpose:**

Diabetic retinopathy (DR) is one of the most important microvascular complications in both type 1 and type 2 diabetes. In Brazil, its proliferative form is the second cause of irreversible blindness among adults of working age. Despite the strong association of DR with disease duration and degree of chronic hyperglycemia, genetic predisposition has been recognized as a possible trigger in the development of this complication. Recent studies have demonstrated that the development of DR in patients with type 1 diabetes is associated with the occurrence of polymorphisms at the 5'-end of the aldose reductase gene (ALR2). There are no reports investigating these polymorphisms in type 1 diabetes Brazilian patients. The aim of this study was to investigate the relationship between the AC(n) repeat and C(-106)T polymorphisms of the *ALR2* gene with the susceptibility to the development of DR in Brazilian patients with type 1 diabetes.

**Methods:**

We selected 64 patients who had diabetes for at least 10 years from Santa Casa de São Paulo and State University of Campinas. The study group was divided into the following: Group 1, patients with no evidence of diabetic retinopathy; group 2, patients with nonproliferative diabetic retinopathy (NPDR); and group 3, patients with proliferative diabetic retinopathy (PDR), confirmed by fundoscopy. The AC(n) microsatellite region was evaluated through polymerase chain reaction (PCR) and automated genotyping and the C(-106)T substitution through polymerase chain reaction/restriction fragment length polymorphism (RFLP).

**Results:**

When each allele of the AC(n) polymorphism was evaluated, the Z allele (24 repeats) was significantly associated with the development of PDR (p=0.014). The C allele of the C(-106)T substitution wasn't associated with the susceptibility to this microvascular complication (p=0.153). When the Z and C allele were concomitantly evaluated regarding their presence or absence a positive correlation was observed for the presence of both alleles and the development of PDR.

**Conclusions:**

In our sample of Brazilian patients with type 1 diabetes, the presence of the AC(n) polymorphism Z allele may be considered a risk factor for the development of PDR. The C allele of the C(-106)T polymorphism, in association with the Z allele, also increased the risk for the development of PDR, but when it was analyzed by itself there was no association with the complication.

## Introduction

Diabetic retinopathy (DR), one of the most important microvascular complications in both forms of diabetes mellitus, has been considered a major cause of visual impairment worldwide [1-4].

Many biochemical mechanisms have been proposed to explain the structural and functional abnormalities associated with overexposure of the vascular tissues to hyperglycemia including advanced glycation end products (AGEs), increase in aldose reductase (AR) activity, free radicals acummulation (reactive oxygen intermediate pathway, ROI), resulting in oxidative stress and protein-kinase C activation (PKC) through diacylglycerol accumulation (DAG). All these pathways, either directly or indirectly, are associated with the production and signaling of vascular endothelial growth factor (VEGF), contributing to the development of neovessels in DR [5,6].

Although diabetes duration and inadequate glycemic control are important risk factors in the development of DR, genetic factors may play a significant role in the pathogenesis of this complication [7]. Some genes have been specifically implicated in the etiology of DR, including the ones associated with glycosylation, immune response, collagen formation, platelet adhesion and aggregation, and the polyol pathway [8].

AR, the first enzyme of the polyol pathway, probably plays an important role in the pathogenesis of the long-term complications of diabetes by means of alterations that result from sorbitol increase and myo-inositol reduction [7]. AR is responsible for the conversion of glucose to sorbitol, which is then converted into fructose by the enzyme sorbitol dehydrogenase (SORD). During the exposition to hyperglycemia, there is an enhanced flux of AR (increased enzyme activity and expression of the aldose reductase gene-*ALR2*) and a reduced flux of SORD. This imbalance results in the accumulation of sorbitol, which leads to several abnormalities in cellular metabolism and hence contributes to the development of diabetic microvascular complications [9,10].

Different reports suggest that the AC(n) dinucleotide polymorphism, located 2.1 kb upstream the transcription start site of *ALR2*, is associated with the susceptibitily to diabetic complications as nephropathy and retinopathy [7,9-12]. Recently Cheung and co-workers, through the study of *ALR2* gene knockout mice, suggested that AR is responsible for the early events in the pathogenesis of diabetic retinopathy, triggering a cascade of retinal lesions that would lead to neovascularization [13].

In addition, another polymorphism, the C-(106)T in the promoter region of *ALR2*, has also been correlated with the susceptibility to the development of DR, acting alone or combined with the AC(n) polymorphism [7,9,10,14].

To the best of our knowledge, there are four studies related to these polymorphisms in *ALR2* in Latin America. Two studies were performed by Olmos and colleagues in Chilean type 2 diabetic patients, who found a positive correlation between the Z-2 allele of the AC(n) polymorphism and a faster progression of retinopathy when compared with patients who harbored other alleles [12] and also an association between the CC genotype of the C(-106)T polymorphism with the development of retinopathy [15]. The first study performed by Santos and co-workers analyzed a specif type 2 diabetic population in the south Brazil, which was denominated Euro-Brazilian. They reported no correlation between the C(-106)T polymorphism with DR [14]. In a recent study the same group evaluated a greater number of Caucasian-Brazilians as well as African-Brazilians DM2 patients, demonstrating that among the Caucasian-Brazilian patients the CC genotype was independently associated with an increased risk of PDR [16].

The aim of this study was to investigate a possible correlation between the AC(n) and C(-106)T polymorphisms and diabetic retinopathy in DM1 Brazilian patients. This population is characterized by an ethnic admixture and there is no study that evaluates these two *ALR2* structural alterations in DM1 patients from Brazil.

## Methods

### Patients

The study group was constituted by 64 Brazilian patients with diabetes mellitus type 1, with at least 10 years of disease. Thirty five were selected in the pediatric and adult endocrinology service, Irmandade da Santa Casa de Misericórdia de São Paulo, and 29 in the adult endocrinology service, State University of Campinas, Unicamp, during a 2 year period, from June 2003 to April 2005. This study adhered to the tenets of the Declaration of Helsinki and received the approval of Ethics Committee of both institutions. All patients signed an informed consent.

Patients underwent a comprehensive clinical and ophthalmic evaluation. The following demographic, clinical and laboratory information was documented for each subject: mean age, sex, diabetes, age of onset, and duration, presence of hypertension (blood pressure was corrected according to sex and age [17]), body mass index Z score (ZBMI; Growth Analyser 3 Software, Version 3.0, Dutch Growth Foundation), presence of chronic complications (neuropathy and proteinuria), fundoscopy, average glycated HbA and insuline dosage.

Ophthalmic evaluation was performed by indirect fundoscopy and biomicroscopy and, in case of abnormalities, documented by retinography. In the presence of diabetic retinopathy, it was classified as non-proliferative diabetic retinopathy (NPDR) and proliferative diabetic retinopathy (PDR) [18]. The following clinical and laboratory parameters were compared according to the status of retinopathy: mean age, diabetes age of onset, duration of disease, ZBMI, presence of hypertension, average glycated HbA and insulin dosage.

### Controls

The normal control group was composed of 55 healthy blood donors who had no family history of diabetes. They were recruited from Hemocentro, Santa Casa de São Paulo, and have an ethnic background and geographic distribution similar to that of the study group.

### AC(n) polymorphism

Genomic DNA was isolated from peripheral leukocyte by standard methods [19]. Amplification of a 138 bp fragment (Z allele), which includes the repetition region AC, located 2.1 kb upstream the *ALR2* gene was performed, using a modification of the method reported by Ko and colleagues [11]. The forward primer was marked at the 5' end with the FAM fluorescent marker (Invitrogen^TM^ Life Technologies, Carlsbad, CA).

After the amplification, 1 μl of the PCR product, diluted 1:7 in ddH_2_O, was mixed with 0.5 μl of the internal molecular weight marker (GeneScan^TM^ 500 ROX^TM^ Size Standard, Applied Biosystems, Warrington, UK) and 23.5 μl of deionized formamide (Hi-Di^TM^ Formamide, Applied Biosystems, Foster City, CA). This mixture was denatured at 95 °C for 2 min and kept on ice until the submission to capillary electrophoresis in the automatic DNA analyzer ABI PRISM 310 (Applied Biosystems), at 60 °C for 20 min per sample. Results were analyzed through the GeneScan software (ABI Prism® GeneScan Analysis Software version 3.7 for Windows NT® Platform, Applied Biosystems) and Genotyper (ABI Prism® Genotyper® 307 NT Software, Applied Biosystems).

In order to confirm the number of repetitions and hence establish a reference for genotyping, patients who presented the alleles in homozygosity were selected and their DNA samples submitted to sequencing reactions, using the following primers: forward 5'-GGA CTA AGG GAA GGC AAG TG-3' and reverse 5'-CCT TCA ATG GAA TCC TCC TG-3' (Genbank accession number U72619). PCR products were directly sequenced through the big dye terminator cycle sequencing ready reaction kit, version 3.1 (Applied Biosystems, Foster City, CA), according to manufacturer's instructions and electrophoresed in a DNA automatic analyzer (ABI Prism 310; Applied Biosystems, Foster City, CA). The sequences were searched for similarities by using the BLAST search algorithm available at BLAST website.

### C(-106)T polymorphism

A fragment of the *ALR2* gene promoter region containing the C(-106)T polymorphism was amplified by PCR. The following pair of primers was selected: Forward-5'-TTC GCT TTC CCA CCA GAT AC-3', between nucleotides -255 and -235 and reverse-5'-CGC CGT TGT TGA GCA GGA GAC-3', between nucleotides 50 and 71, resulting in the amplification of a 326 bp fragment. Samples were subjected to 35 cycles of amplification, each consisting of denaturation at 95 °C for 1 min, annealing at 57 °C for 1 min, and extension at 72 °C for 1 min with a final extension at 72 °C for 7 min (GeneAmp® PCR System 9700, Applied Biosystems) [7,20].

In order to detect the C(-106)T polymorphism, the 326 bp fragment was digested with the restriction endonuclease *Bfa*I (New England BioLabs, MA), once this substitution creates a new restriction site to the enzyme. The homozygous CC individuals presented two fragments of 234 bp and 92 bp; the homozygous TT individuals presented three fragments of 175 bp, 92 bp, and 59 bp and finally, the heterozygous CT individuals presented four fragments of 234 bp, 175 bp, 92 bp, and 59 bp [7].

### Statistical analysis

The descriptive analysis of demographic, clinical, diagnosis and molecular data of our patients was performed.

Based on fundoscopy findings, patients were divided into NORMAL and DR. Genotypes and allelic frequency of the AC(n) and C(-106)T polymorphisms were compared according to the following stratifications: 1. Normal X DR, 2. Normal X NPDR X PDR, 3. Normal X PDR, and 4. NPDR X PDR.

The data were loaded in the Statistical Program for the Social Sciences (SPSS, version 13.0, SPSS Inc., Chicago, IL). Either the Χ-square test or the Fisher's exact test was used for categorical variables. The Mann-Whitney Test was used for continuous variables. p-values <0.05 were considered to be statistically significant.

## Results

The mean age of the studied patients was 26.72±10.52 years, ranging from 12.10-57 years. Forty-three patients were female (67.2%) and, in relation to the skin color, 47 were white (73.4%), five black (7.8%), and 12 mulatto (18.8%). As stated at both introduction and discussion, the Brazilian population is highly miscigenated, making it difficult to establish an ethnicity. In this study, skin color was arrived at based on the observation of the researchers. The classification of white, black, and mulatto was determined not only by the skin color, but also by the observation of other characteristics, such as hair, lips, and nose shape. The predominant genotype for the AC(n) polymorphism was Z/Z-2 (26.6%), followed by Z/Z+2 (12.5%), and Z-2/Z-2 (12.5%), totalizing 18 different genotypes observed. The most common alleles of this polymorphism were Z-2 (35.2%), Z (35.2%), and Z+2 (15.6%). The distribution of the alleles of the AC (n) polymorphism in relation to the classification of fundoscopy as well as in normal controls is depicted in Table 1. The most common genotype of the C-106T polymorphism was CT corresponding to 50% of the cases, followed by CC, corresponding to 40.3% of the analyzed cases. In relation to the distribution of the alleles of this polymorphism, allele C, encompassing 65.3% of the cases, was more frequent than allele T (34.7%).

**Table 1:** Allelic frequency of AC(n) repeats in diabetic patients and normal controls.

	Diabetes mellitus type 1 patients			
	Normal (%)	NPDR (%)	PDR (%)	Controls
Allele	n=66	n=32	n=30	(%) n=110
Z+12	0 (0.0)	0 (0.0)	0 (0.0)	2 (1.8)
Z+6	2 (3.0)	1 (3.1)	0 (0.0)	2 (1.8)
Z+4	4 (6.1)	1 (3.1)	1 (3.3)	7 (6.4)
Z+2	13 (19.7)	5 (15.6)	2 (6.7)	8 (7.3)
Z	20 (30.3)	8 (25.0)	17 (56.7)	48 (43.6)
Z-2	23 (34.8)	14 (43.8)	8 (26.7)	38 (34.6)
Z-4	3 (4.5)	3 (9.4)	1 (3.3)	2 (1.8)
Z-6	1 (1.5)	0 (0.0)	1 (3.3)	1 (0.9)
Z-12	0 (0.0)	0 (0.0)	0 (0.0)	1 (0.9)
Z-16	0 (0.0)	0 (0.0)	0 (0.0)	1 (0.9)

NPDR represents nonproliferative diabetic retinopathy; PDR represents proliferative diabetic retinopathy; n represents number of alleles.

The frequencies of both AC(n) and C(-106)T genotypes in our normal control population were in Hardy-Weinberg equilibrium. Ten alleles were observed for the AC(n) polymorphism, ranging from Z-16 to Z+12, with Z (43.6%) and Z-2 (34.6%) being the most frequent ones. For the C(-106)T polymorphism, allele C was present in 67.9% of the subjects and allele T in 32.1%.

Among the clinical and laboratory parameters evaluated, only age and diabetes' duration were different between patients with and without DR. Both parameters were higher in the group with DR. Average patients age was 24.16±9.59 years in the group without DR and 29.44±10.93 years in the group with DR (p=0.036). Duration of diabetes was 14.43±3.81 years in patients without DR and 17.87±6.28 years in patients with DR (p=0.015).

### AC(n) polymorphism

No association was observed between the genotypes of the AC(n) polymorphism and DR development, when these were evaluated according to the groups' stratifications. On the other hand, when each allele was evaluated in relation to fundoscopy, only the Z allele appeared to be significantly associated with the presence of PDR, with a risk greater than three times for individuals harboring this allele (Normal X PDR, risk = 3.01 and NPDR X PDR, risk=3.92). There was no difference regarding the classification Normal X DR (p=0.170). Table 2 and Table 3 show patients distribution according to the presence or absence of the Z allele and the development of PDR. There was no difference in the duration of the disease in relation to the presence or absence of the Z allele (p=0.053). Once the Z allele was associated with the development of PDR, combined genotypes including this allele (Z) and any other allele with exception to Z (X) were compared in eye fundoscopy results (ZZ x ZX, ZZ x XX, and ZX x XX), confirming the susceptibility to the development of this complication in ZZ individuals in relation to XX individuals (Table 4). The average duration of disease for patients with the ZZ, ZX and XX alleles was 14.43±3.95, 17.79±6.07, and 16.10±5.40, respectively.

**Table 2:** Evaluation of the of the AC(n) polymorphism Z allele in relation to diabetic retinopathy, based on the classification normal and proliferative diabetic retinopathy.

	Fundoscopy classification	
Allele Z	PDR (%) n=30	Normal (%) n=66
Present	17 (56.7)	20 (30.3)
Absent	13 (43.3)	46 (69.7)

Χ-square test: p=0.014; odds ratio=3.01 (1.23-7.34, confidence interval=95%). PDR represents proliferative diabetic retinopathy n represents number of alleles=96.

**Table 3:** Evaluation of the of the AC(n) polymorphism Z allele in relation to diabetic retinopathy, based on the classification of nonproliferative diabetic retinopathy and proliferative diabetic retinopathy.

	Fundoscopy classification	
Allele Z	PDR (%) n=30	NPDR (%) n=32
Present	17 (56.7)	8 (25.0)
Absent	13 (43.3)	24 (75.0)

Χ-square test: p=0.011 odds ratio=3.92 (1.34-11.53, confidence interval=95%). PDR represents proliferative diabetic retinopathy, NPDR represents nonproliferative diabetic retinopathy, and n represents number of alleles=62.

**Table 4:** Distribution of patients without retinopathy and with proliferative diabetic retinopathy, according to the AC(n) polymorphism ZZ genotype.

	Fundoscopy classification	
Genotype	PDR (%) n=6	Normal (%) n=17
ZZ	4 (66.7)	2 (11.8)
XX	2 (33.3)	15 (88.2)

X represents any allele other than Z. Fisher's exact test is p=0.021.

### C-(106)T polymorphism

In relation to the C(-106)T polymorphism, we found no association between genotypes and the susceptibility to the development of DR (p = 0.316). However, our data show that among the 14 patients with PDR, no one presented with the TT genotype (Table 5). Also, when the alleles were analyzed, we observed no correlation between these and susceptibility or protection to the development of the disease (p=0.153).

**Table 5:** Distribution of patients without retinopathy, with nonproliferative diabetic retinopathy, and with proliferative diabetic retinopathy, based on the genotypes of the C(-106)T polymorphism.

	Fundoscopy classification		
Genotype	Normal (%) n=33	NPDR (%) n=15	PDR (%) n=14
CC	10 (30.30)	8 (53.33)	7 (50.00)
CT	18 (54.54)	6 (40.00)	7 (50.00)
TT	5 (15.16)	1 (6.67)	0 (0.00)

Χ-square test: p=0.316. NPDR represents nonproliferative diabetic retinopathy; PDR represents proliferative diabetic retinopathy; n represents number of patients=62.

### Haplotype AC(n)/C(-106)T

When patients were analyzed in relation to the presence of the C and Z alleles concomitantly and the development of DR, it was observed that the presence of both alleles (allele C + allele Z) represents a tendency to the development of PDR (Normal X PDR, risk of PDR=5.65; Table 6). However, when we analyzed the presence of C and Z alleles versus only C (p=0.159) and C and Z alleles versus only Z (p=1,000), we found no statistically significant differences between the groups.

**Table 6:** Distribution of patients without retinopathy and with proliferative diabetic retinopathy based on the presence of the C allele of the C(-106)T polymorphism and the Z allele of the AC(n) polymorphism.

	Fundoscopy classification	
Allele C + Allele Z	PDR (%) n=14	Normal (%) n=33
Present	12 (85.7)	17 (51.5)
Absent	2 (14.3)	16 (48.5)

Χ-square test: p=0.027, odds ratio=5.65 (1.09-29.26, confidence interval=95%). PDR represents proliferative diabetic retinopathy; n represents number of patients=47.

## Discussion

It is intriguing that despite good control of blood glucose levels, some diabetic patients develop complications such as microangiopathies while others remain problem free after a long period of diabetes. Today there is considerable evidence that some diabetic complications are influenced not only by the long exposure to hyperglycemia but also by genetic factors [8,9,11,12,21].

Aldose reductase gene promoter polymorphisms AC(n) and C(-106)T have been extensively investigated in different populations. Some studies have shown that they may lead to increased mRNA expression of the *ALR2* gene and consequently to the accumulation of sorbitol and cellular damage [21,22].

The most common genotype related to the AC(n) polymorphism is represented by 24 dinucleotides AC and denominated Z [9,11,23,24]. Although results conflict, depending on the population, most studies correlate the Z-2 allele with complication risk while the Z+2 allele appears to be linked to protection [7,9-12].

In our population, the Z allele appeared to be significantly associated with the development of PDR, with a complication development risk that was three times greater than individuals harboring other alleles. This data differ from all other groups previously studied. It is important to consider that our population is ethnically distinct and that there are no previous studies of this polymorphism in the Brazilian population. In Japanese patients with type 2 diabetes the Z-4 allele may determine the predisposition to retinopathy [25] while Wang and coworkers [10] observed that the Z+6 allele may confer a protective effect. In order to evaluate the existence of a possible bias due to the greater duration of diabetes in individuals with diabetic retinopathy, we evaluated the duration of the disease in the presence and absence of the Z allele. Our analysis showed that this variable is not statistically different between patients with and without this allele. However, the p value is too close to the limit of significance and hence the duration of the disease could not be excluded as a confounding factor. In contrast, the duration of diabetes was greater among patients with the XX genotype (16.10±5.40) than in individuals with the ZZ genotype (14.43±3.95).

A more definite relationship can be established between the Z allele of the AC(n) polymorphism and predisposition to the development of PDR, by performing gene expression studies in our population of DM1 patients, as the study conducted by Shah and colleagues [22].

In the analysis of the genotype of our patients we did not find correlation with the susceptibility or protection to DR development, probably due to the small number of patients for each genotype, as reproduced by previous studies [12,24,26].

In respect to the C(-106)T polymorphism, many groups suggest that the presence of the T allele gives some protection to the development of DR, and the presence of the C allele raises the susceptibility to the development of this complication [7,9]. In relation to the functional aspect, Li [21] examined the possible correlation between the C-(106)T polymorphism and the *ALR2* gene expression, and found that the presence of the C allele increases twice mRNA expression.

In the present study, it was not observed any statistical association based on the genotypes, although no patient with PDR presented the TT genotype and among 6 individuals with this genotype, 5 were normal and 1 presented a mild form of DR. The C allele was not related to DR susceptibility when analyzed separately, probably due to the size of our sample. Our findings are similar to the ones described by Santos for DM2 patients [14].

Kao [7] and Demaine [9] analyzed the (AC)n and C(-106)T polymorphisms and verified that the C allele in association with the Z-2 allele is related to the development of DR. When we investigated the effect of the presence and absence of both polymorphisms in our population, we found a 5.65 times greater tendency for the development of PDR to individuals who presented both alleles compared to the ones who didn't present. We found no increased risk in the development of PDR when the presence of C and Z alleles was compared to the presence of only C or only Z allele.

There are many factors that could explain the association of the Z allele with PDR in our population. First, populations evaluated for these polymorphisms are ethnically distinct [7,9-12,25]. Second, the Z allele could be linked to another susceptibility gene, acting as a modulator, or even it could be linked to another polymorphisms within the same gene as C(-106)T, for example. Finally, the complex mechanism of the development of retinal damage, involving at least four different metabolic pathways, suggests that we could possibly have a genotype pattern in our population that leads the Z allele in the *ALR2* gene to become associated with the development of PDR [27].

One possible source of bias in case-control studies is population stratification. The Brazilian population in this particular set of patients has a high degree of admixture, hindering accuracy in any attempt to stratify allele frequencies according to ethnic backgrounds. However, we have to address that both the control and the study groups are similar in regard to ethnical background and geographical distribution. Besides, allele frequencies for both polymorphisms are in Hardy-Weinberg equilibrium in the normal control group. The study design, as well as the sample size, could also interfere with the results.

In conclusion, this is the first report regarding the investigation of *ALR2* promoter polymorphisms in DM1 Brazilian patients. Two other studies were performed in DM2 Brazilian patients, evaluating only the C(-106)T substitution. The results demonstrate an association of the Z allele of the AC(n) polymorphism, alone or in combination with the C allele of the C(-106T) polymorphism, with the predisposition to the development of PDR. This work opens possibilities to further functional studies in order to confirm the influence of this genetic change in the susceptibility to diabetic microvascular complications, which are among the main causes of visual impairment in the world. This kind of study is also important to establish the role of genetic factors in the risk of developing DR, improving the control of the disease.
